# LTR retroelements in the genome of *Daphnia pulex*

**DOI:** 10.1186/1471-2164-11-425

**Published:** 2010-07-09

**Authors:** Mina Rho, Sarah Schaack, Xiang Gao, Sun Kim, Michael Lynch, Haixu Tang

**Affiliations:** 1 School of Informatics and Computing, Indiana University, Bloomington, IN 47405, USA; 2 Department of Biology, University of Texas-Arlington, Arlington, TX 76019, USA; 3 Department of Biology, Indiana University, Bloomington, IN 47405, USA; 4 Genomics and Bioinformatics, Indiana University, Bloomington, IN 47405, USA

## Abstract

**Background:**

Long terminal repeat (LTR) retroelements represent a successful group of transposable elements (TEs) that have played an important role in shaping the structure of many eukaryotic genomes. Here, we present a genome-wide analysis of LTR retroelements in *Daphnia pulex*, a cyclical parthenogen and the first crustacean for which the whole genomic sequence is available. In addition, we analyze transcriptional data and perform transposon display assays of lab-reared lineages and natural isolates to identify potential influences on TE mobility and differences in LTR retroelements loads among individuals reproducing with and without sex.

**Results:**

We conducted a comprehensive *de novo *search for LTR retroelements and identified 333 intact LTR retroelements representing 142 families in the *D. pulex *genome. While nearly half of the identified LTR retroelements belong to the *gypsy *group, we also found *copia *(95), BEL/*Pao *(66) and DIRS (19) retroelements. Phylogenetic analysis of reverse transcriptase sequences showed that LTR retroelements in the *D. pulex *genome form many lineages distinct from known families, suggesting that the majority are novel. Our investigation of transcriptional activity of LTR retroelements using tiling array data obtained from three different experimental conditions found that 71 LTR retroelements are actively transcribed. Transposon display assays of mutation-accumulation lines showed evidence for putative somatic insertions for two DIRS retroelement families. Losses of presumably heterozygous insertions were observed in lineages in which selfing occurred, but never in asexuals, highlighting the potential impact of reproductive mode on TE abundance and distribution over time. The same two families were also assayed across natural isolates (both cyclical parthenogens and obligate asexuals) and there were more retroelements in populations capable of reproducing sexually for one of the two families assayed.

**Conclusions:**

Given the importance of LTR retroelements activity in the evolution of other genomes, this comprehensive survey provides insight into the potential impact of LTR retroelements on the genome of *D. pulex*, a cyclically parthenogenetic microcrustacean that has served as an ecological model for over a century.

## Background

Transposable elements (TEs) have been found in most eukaryotic genomes and often constitute a significant portion of the genome (e.g., 80% of maize [1], 45% of human [2], and 5.3% of the fruit fly genome [3,4] are known to be comprised of TEs). Because they can transpose from one location to another within the genome or across genomes, the identification of TEs and analysis of their dynamics are important for a better understanding of the structure and evolution of both genomes and TEs themselves [5,6]. Based on the mechanism of transposition, TEs are categorized into two major classes. The elements in class I (retroelements) are transposed through reverse transcription of an RNA intermediate, whereas the elements in class II (DNA transposons) are transposed through a cut-and-paste transposition mechanism [6]. LTR retroelements, one type of class I retroelements, are characterized by long terminal repeats (LTRs) at their 5' and 3' ends, and encode genes required for their retrotransposition (e.g., *gag *and *pol*). In several species, LTR retroelements have amplified to high levels resulting in major modifications of the host genome (e.g., in rice [7,8])

In order to identify LTR retroelements in whole genome sequences, many computational methods have been developed [9]. *De novo *approaches search for putative pairs of LTRs in the genome [10,11]. The identified LTRs can then be combined with other important sequence features, including target site duplications (TSDs) and conserved protein domains, to identify intact LTR retroelements. Once the intact LTR retroelements are found, homology-based searching (e.g., using RepeatMasker with a library of intact LTR retroelement sequences) can be used to identify additional fragmented elements and solo LTRs in the genome.

Although newly-sequenced genomes may contain many TEs, it is often unclear what proportion of the identified elements remains active in the population. Recent advances in tiling array technology provide opportunities for measuring gene transcription levels at a genome-wide scale, which can also be used to detect the activity of the TEs that are identified *in silico*. Even though transcription of TEs is not sufficient to cause their transposition, it is a necessary first step for mobilization of retroelements. In addition, recent work suggests transposable elements may upregulate expression of host genes [12] or, more generally, that TEs may function as part of genome-wide regulatory networks [13]. Because transcription patterns of TEs are known to vary under different environmental conditions and/or at developmental stages, analysis of transcription profiles is the first step towards understanding what factors might induce mobilization of TEs in the host genome.

Transposon display can be used to compare differences in TE load among individuals or populations over time or from different regions. One of the features of the host genomic environment that has been proposed to significantly impact TE mobility and distribution is the frequency of recombination [14,15]. Because *D. pulex *is a cyclical parthenogen, it is possible to assess the role of recombination in TE proliferation in this species without many of the confounding variables that have plagued past comparisons (e.g., species differences [16]). This is because natural populations of *D. pulex *are known to lose the ability to reproduce sexually (thereby becoming obligate asexuals) and sexual reproduction can be suppressed or promoted by manipulating laboratory conditions. Thus, it is possible to use this system to look more closely at the short-and long-term impact of recombination on TE abundance by combining laboratory and field comparisons.

The analysis of *D. pulex *presented in this paper represents the first such data for a freshwater aquatic arthropod and cyclical parthenogen and provides an opportunity to better understand the dynamics of TEs via comparison with other well-studied systems. LTR retroelements have been shown to exert a strong impact on the genome of other organisms (see [17] for a recent review) and may be capable of similar mobility and influence in this species as well.

## Results

### Identification of LTR retroelements in the *Daphnia *genome

Intact LTR retroelements were identified using multiple empirical rules: similarity of a pair of LTRs at the both ends, the structure of internal regions (IRs), di(tri)-nucleotides at flanking ends, and TSDs. The definition of *intact *LTR retroelement was adapted from previous studies [3,18,19], and is limited to those that encode protein domains such as *gag *and *pol *and have pairs of LTRs at both ends. The intact elements identified were clustered into families based on the sequence similarity of LTRs between elements (> 80%). The program MGEScan-LTR [11] identified 333 intact LTR retroelements in the *D. pulex *genome and clustered them into 142 families (Table 1). The identified elements include 66 BEL, 95 *copia*, 19 DIRS, and 153 *gypsy *elements, which were clustered into 26, 44, 16, and 56 families, respectively (Table 1 and Additional file 1 Table S1). Among these, 251 elements have a pair of tri-nucleotides (TGT/ACA) flanking the ends of the LTRs and TSDs ranging from 4 to 6 bp in length.

**Table 1:** Summary of LTR retroelements in *D. pulex*.

Group	# of elements (families)	Avg. Length of LTR (bp) (min - max)	Avg. Length of elements (bp) (min - max)
BEL	66 (26)	441 (193 - 1735)	7350 (3349 - 12536)
*copia*	95 (44)	288 (172 - 602)	5164 (4064 - 8184)
DIRS	19 (16)	119 (88 - 170)	4850 (4313- 5501)
*gypsy*	153 (56)	354 (134 - 938)	7645 (4026 - 12862)

In order to understand how the LTR retroelements in the *D. pulex *genome are different from those in other invertebrate genomes, we applied MGEScan-LTR [11] to four additional genomes: *Anopheles gambiae*, *Bombyx mori*, *Drosophila melanogaster*, and *Oryza sativa*. Although these genomes have been analyzed in previous studies [3,18,20,21], we searched for the intact LTR retroelements following the same procedure used for *D. pulex *(Additional file 1 Table S2). The elements that we identified using our pipeline largely overlap with previously described elements for each species. Small differences might be due to the difference between the versions of genomic sequences and/or the criteria used in these analyses.

### Distribution of LTR retroelements in different groups

**Figure 1:**
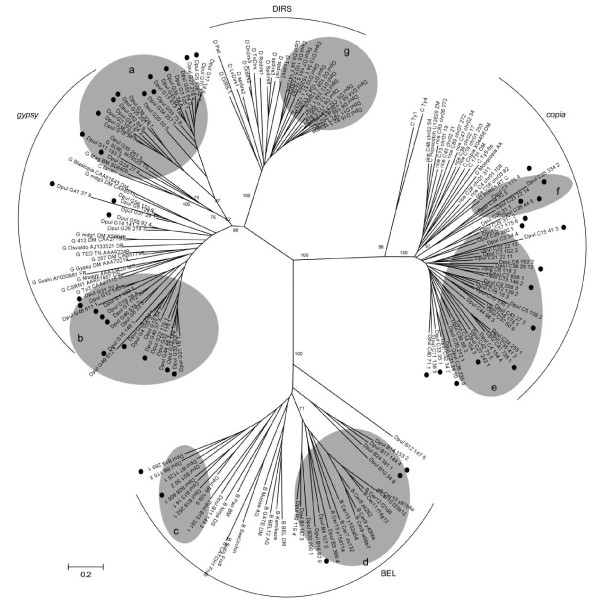
**Neighbor-joining phylogenetic tree of LTR retroelements in the *D. pulex *genome**. The RT sequences are from the newly-identified elements (an element from each family) in the *D. pulex *genome along with previously known retroelements; (a and b) the elements in *gypsy *group, (c and d) the elements in BEL/Pao group, and (e and f) the elements in *copia *group, and (g) the elements in DIRS group. *D. pulex *retroelements were named after the family name and element name without underscores. For example, in Dpul B1 104 3, Dpul B1 is the family name and 104 3 is the element name. Previously known retroelements follow their annotated names such as *SURL *and *Maggy*. The bootstrap value for main branches and a divergence scale are indicated. The families that have transcriptionally active elements are marked with black circles. The *D. pulex *elements identified are marked in gray circles.

To date, *gypsy *is the most abundant among the four main groups of LTR retroelements (*gypsy*, *copia*, BEL, and DIRS) in invertebrate genomes such *D. melanogaster*, *B. mori*, and *A. gambiae *[3,21]. In particular, the *gypsy *elements in the *D. melanogaster *genome belong to one of three main lineages *Gypsy, Mdg1*, and *Mdg3 *[22], whereas the elements in the *A. gambiae *genome belong to five distinctive lineages, including two additional lineages, *CsRN1 *and *Mag *[23]. A total of 153 intact *gypsy *elements from 56 families were identified in the *D. pulex *genome, which corresponds to 46% of all intact LTR retroelements identified in this study (Table 1). The phylogenetic analysis of the reverse transcriptase (RT) sequences from these elements revealed that they consist of two major subgroups. One has high sequence similarity to the *Mag *lineage, but the other is distant from any known major gypsy lineages (Figure 1). Among the 22 families in the first subgroup, the neighbor-joining tree shows that the family *Dpul_G24 *is close to the *Mag *element (bootstrap value of 81), whereas *Dpul_G35 *and *Dpul_G11 *are close to the *SURL *element (bootstrap value of 62). Notably, the elements in *Dpul_G24 *family are closest to the *Mag *elements (BLAST E-value ~ 0.0) found in *Chlamys farreri*, which contain only a single open reading frame (ORF) encoding both *gag *and *pol *proteins.

**Figure 2:**
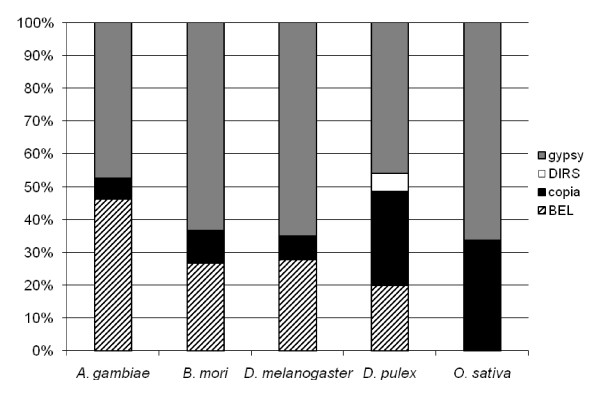
**Composition of BEL, *copia*, DIRS, and *gypsy *elements**. The percentage of intact LTR retroelements in each group is shown for the *D. pulex *genome along with four other genomes (*A. gambiae*, *B. mori*, *D. melanogaster*, and *O. sativa*).

*Copia *elements are known to be more abundant in plant genomes than animal genomes (e.g., 37 out of 57 families in the *O. sativa *genome) [18]. Only a small number of *copia *elements have been identified in invertebrate genomes [3,22,24], including the *copia *and *1731 *families in the *D. melanogaster *genome, and the *Mosqcopia *elements in the *Aedes aegypti *genome. Interestingly, our results indicate that the *copia *group is highly abundant and diverse in the *D. pulex *genome. A total of 95 intact *copia *retroelements (clustered into 44 families) were identified, which constitute 29% of all intact LTR retroelements identified in the *D. pulex *genome. The ratio of the numbers of intact *copia *to *gypsy *elements is 0.62, which is very high compared with other insect genomes (0.11 for *D. melanogaster *and 0.13 for *A. gambiae*; Figure 2). The RT sequences from the *D. pulex *retroelements and some representative elements from other genomes (*1731 *and *copia *from *D. melanogaster*, *RIRE1 *and 11 additional LTR retroelements from *O. sativa*, *Hopscotch *from *Zea mays*, and *Ty1 *and *Ty4 *from *Saccharomyces cerevisiae*) were used in the phylogenetic analysis. *D. pulex copia *elements were roughly clustered into two subgroups. One subgroup consists of four elements (bootstrap value of 100), and the other subgroup consists of the remaining elements (Figure 1).

The *D. pulex *genome has fewer BEL elements compared with other insect genomes for which data exist (*D. melanogaster *and *A. gambiae*), which have more BEL elements than *copia *elements (Figure 2). A total of 66 intact BEL retroelements were identified and clustered into 26 families, which correspond to 20% of all intact LTR retroelements found in this genome. The BEL/*Pao *retroelements are known to have four major lineages: *Cer*, *Pao/Ninja*, *Bel/Moose*, and *Suzu *[25-29]. Six BEL families identified in the *D. pulex *genome were close to the *Cer *retroelements from *C. elegans *in the neighbor-joining tree (bootstrap value of 87, Figure 1). The other 20 BEL families in the *D. pulex *genome were close to the *Pao/Ninja *lineage.

DIRS retroelements typically contain inverted repeats instead of direct repeats, and are typically much shorter than classic LTRs [30,31]. Hence, we modified MGEScan-LTR program accordingly to search for proximal inverted repeats and ORFs encoding proteins such as RT and tyrosine recombinase (YR). A total of 19 intact DIRS retroelements (from 16 families) were identified in the *D. pulex *genome, which correspond to 6% of all elements identified in this genome. Given that no DIRS element has been identified in any previously surveyed arthropod genome except *Tribolium castaneum *[30], *D. pulex *has the largest number of DIRS elements among the arthropods so far.

### Transcriptional activity of LTR retroelements

**Figure 3:**
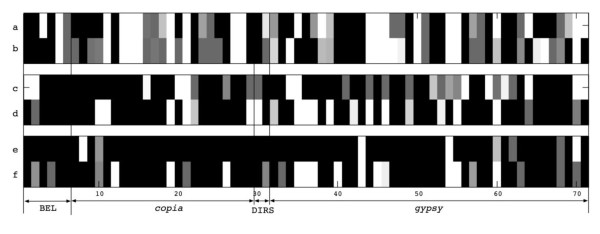
**Expression map showing transcriptional activity**. Each row is from different condition: female (a), male (b), control for metal exposure (c), metal exposure (d), control for kairomone exposure (e), and kairomone exposure (f). Each column represents individual LTR retroelements (Additional file 1 Table S3). The log intensity value ranges from 0.0 (in black) to 4.82 (in white).

The first step of the transposition of LTR retroelements is transcription. The transcribed elements are then reverse transcribed into DNA and inserted into the host genomes by themselves or with help of other autonomous elements. However, all the transcribed elements are not necessarily transposed into host genomes. Therefore, the analysis of transcriptional activity can help assess the potential mobility of the LTR retroelements. We used expression tiling array data sets from six separate experimental conditions for our analysis (Colbourne et al. *manuscript in preparation*). The transcriptome of adult females was compared to that of adult males to assess sex-based differences in LTR retroelement activity (Figure 3a and 3b). The transcriptome of mature stage-specific female animals exposed to metals was compared to similar stage reference samples to assess human-induced environmental stress conditions (Figure 3c and 3d). Finally, the transcriptome of 4^th ^instar juvenile females exposed to predator kairomones from the dipteran larvae *Chaoborus *was compared to similar stage reference samples to assay transcription levels under natural environmental stress conditions (Figure 3e and 3f). Transcriptionally-active regions (TARs) on the tiling array were observed across the entire genome (data are available at http://insects.eugenes.org:8091/gbrowse/cgi-bin/gbrowse/daphnia_pulex8). We located overlapping regions between the TARs and all 333 LTR retroelements identified in this study to determine the transcription levels of the corresponding elements (Additional file 1 Table S3 and S4). In total, 71 elements overlap with at least one of the TARs, including 6 BEL, 23 *copia*, 2 DIRS, and 40 *gypsy *elements. A similar ratio of transcriptionally active *copia *to *gypsy *elements (0.57) was observed relative to the ratio of total number of *copia *to *gypsy *elements in the whole genome sequence (0.62). Eleven families (*Dpul_C33, C7, C8, G1, G12, G28, G31, G32, G5, G56*, and *G8*) consisting of more than one element overlapped with the TARs.

Among 71 elements with detectable transcriptional activity, 12 elements show long TARs (> 500 bp), as listed in Table 2. The element *Dpul_C40 *shows very long TARs (85% of the element length) with high expression level (log2 intensity of 5.37) in the adult female data set. Interestingly, the elements *Dpul_C17 *and *Dpul_C28*, both show significant transcriptional activity (log2 intensity of 4.78 for both elements) and long TARs (1453 bp for *Dpul_C17*, and 1066 for *Dpul_C28*), but have relatively low LTR similarities (92.5% and 92.7%, respectively). Pairwise alignment of their LTR pairs showed that the relatively low similarities were due to a short fragment (~20 bp) inserted in one of the LTR sequences. Therefore, these elements might still represent a recent insertion, and remain transcriptionally active in the natural *D. pulex *population since low similarity is mainly caused by the insert of single short fragment instead of several accumulated mutations.

**Table 2:** LTR retroelements overlapping with transcriptionally active regions.^1^

Family	Scaffold	Start	Element length (bp)	Total length of TARs (bp)	Expression level^2^	Similarity of LTRs (%)^3^
*Dpul_B25*	48	724286	3577	1557	3.84	97.4
*Dpul_C7*	836	2600	5916	1150	0.58	99.3
*Dpul_C8*	40	919844	5282	1757	4.13	98.4
*Dpul_C8*	118	81961	5289	2171	3.16	99.7
*Dpul_C15*	161	267301	4681	576	0.73	100.0
*Dpul_C17*	175	143408	5473	1453	4.78	92.5
*Dpul_C28*	260	73082	5837	1066	4.78	92.7
*Dpul_C33*	35	412714	4633	828	0.58	100.0
*Dpul_C40*	71	404089	4744	4041	5.37	99.5
*Dpul_G7*	169	162521	7344	888	3.95	99.2
*Dpul_G31*	22	74408	13211	536	3.61	99.1
*Dpul_G31*	49	697716	13403	947	2.97	100.0

^1 ^Total length of transcriptionally active regions (TARs) > 500 bp.

^2 ^Maximum value of median intensity in each TAR; the value is log (intensity).

^3 ^Similarity measured between LTRs in each element.

Among the three experiments under different conditions, *Dpul_G5 *and *Dpul_G7 *showed transcriptional activity across all six different conditions. On the other hand, 20 elements were expressed in only one of the conditions. The expression pattern of these LTR retroelements is shown for each condition (Figure 3). The elements showed higher overall transcriptional activity in the dataset of adults, including female and male (Figure 3a and 3b) than in the other two data sets (mature stage-specific and 4^th ^instar juvenile). In the kairomone-exposed condition, more elements were transcribed than in the control set (Figures 3e and 3f).

### Transposable element dynamics in lab-reared lines and natural populations

In order to assess the role of reproductive mode in retroelement distribution and abundance among sexually-and asexually-reproducing isolates, we developed a transposon display assay for two families of DIRS elements identified in the *D. pulex *genome. We chose DIRS elements because they exhibited intact open-reading frames (which are thought to be a prerequisite for potential activity) and were low-copy number (perhaps making them less likely targets for silencing and readily quantifiable using transposon display; see methods for details). We surveyed mutation-accumulation (MA) lines of *D. pulex *to try and identify if there was any detectable activity and if patterns differed among lines where sex was promoted or prohibited. In addition, we compared TE loads for these two families of retroelements among natural populations in which sex occurs annually (cyclical parthenogens), and in which it does not occur (obligate asexuals).

In mutation-accumulation lines, no germline gains were detected in either retroelement family assayed in the MA lines, but putative somatic gains occurred regularly in both treatments (more often in sexuals than asexuals for the *Dpul_D16 *family; Table 3). Rates of loss were higher in sexuals than in asexuals in the family for which any losses were observed (*Dpul_D5*; Table 3), but losses were not randomly distributed across loci. Instead, they occurred at a subset of the loci scored (4 of 7), presumably those that were heterozygous for the insertion at the beginning of the experiment. The average number of losses at these "high loss" loci was 10, which is very close to the number that would be predicted simply based on segregation of chromosomes and the probability of loss for heterozygous insertions given the sample size of sexual lines surveyed here (11.5 predicted losses when n = 46).

**Table 3:** Rate of loss (per element per generation) and putative somatic gains (per element) observed in two families of transposable elements across mutation-accumulation lines of *D. pulex *where sex was promoted and prohibited (means, SE, t-statistic [*t*] and probability values [*P*] reported).

Element	n	No. of scored sites	Sexuals	Asexuals	*t*	P
**Losses**						
*Dpul_D16*	93	4	0	0	n/a	n.s.
*Dpul_D5*	92	7	0.0028 (± 0.0004)	0.00031 (± 0.0001)	5.44	< 0.000001
**Putative Somatic Gains**						
*Dpul_D16*	93	4	0.0036 (± 0.0003)	0.0016 (± 0.0001)	2.26	0.013
*Dpul_D5*	91	7	0.0020 (± 0.0005)	0.0022 (± 0.0004)	-0.23	0.41

In natural populations, the same two families of DIRS retroelements were surveyed among isolates where sex occurs at least yearly (cyclical parthenogens) and where sex has been completely lost (obligate asexuals). Mean copy number did not differ between cyclical parthenogens and obligate asexuals for *Dpul_D16 *but did for *Dpul_D5*, with copy number in cyclicals exceeding that in asexuals almost threefold (Table 4). In addition to higher loads in sexuals, *Dpul_D5 *also exhibited higher insertion site polymorphism among isolates from sexually-reproducing populations compared to obligate asexuals (with 26 polymorphic loci among cyclical parthenogens vs. only 17 among obligate asexuals). Unlike the pattern observed in DNA transposons (Schaack et al. *accepted*), for the DIRS elements we observed a higher number of singletons (loci occupied in only a single isolate) in cyclically-parthenogenetic isolates relative to obligate asexuals (for *Dpul_D5 *only; 17 versus 13).

**Table 4:** Mean number of occupied sites (± SE) for two families of retroelements assayed across natural populations of *D. pulex*. ^1^

Family	Total no. of occupied sites (*across all isolates*)	Mean no. of occupied sites per isolate		Range	t	*p*
		Cyclicals	Obligates			
*Dpul_D16*	89	1.95 (± 0.2)	2.09 (± 0.2)	0 - 6	-0.5	0.309
*Dpul_D5*	40	1.41 (± 0.2)	0.48 (± 0.1)	0 - 5	4.22	0.00003

^1 ^n = 41 cyclically parthenogenetic isolates, 44 obligate asexual isolates; df = 83.

## Discussion

### Composition of *D. pulex *LTR retroelements

In this study, we have identified 333 intact LTR retroelements in the *D. pulex *genome which were clustered into 142 families. With the library of intact elements identified, 3774 LTR retroelements were found by using Repeatmasker. These retroelements constitute 7.9% of the *D. pulex *genome, which is much higher than *D. melanogaster *(2.6% of 120 Mb genome) [3] and lower than that found in *B. mori *(11.8% of 427 Mb genome) [21]. These levels are all, however, much lower than those found in plants which are known to typically have a much higher proportion of LTR retroelements in their genomes (e.g., 17% in *O. sativa *[18]). In addition to quantifying the LTR retroelement content, our survey showed that the families of LTR retroelements in *D. pulex *are more divergent than previous whole genome analyses have shown. For example, while only 26 *copia *elements were identified in *D. melanogaster *[3], in *D. pulex *there are 95 families (Additional file 1 Table S1; Figure 2). In all invertebrate genomes surveyed in this study, the number of *copia *families are very low (Additional file 1 Table S2), which is also consistent with previous studies [3,21]. Our study also confirmed the presence of 19 DIRS elements in the *D. pulex *genome, which is much higher than any other invertebrate genomes sequenced so far. Only a few DIRS elements have been found in *T. castaneum *[30], *Dictyostelium discoideum*, and some fish (e.g., *Danio rerio *[31]), but none have been identified in the model organisms *D. melanogaster*, *A. gambiae*, and *O. sativa*.

### Survey of transcriptional activities in LTR retroelements

Since transcription of the LTR retroelements is the first step required for their transposition, genome-wide screening of transcriptional data was used to determine what proportion of the LTR retroelements might be active. Tiling arrays use unbiased probes, in contrast to cDNA microarrays which are designed to target gene expression alone, thus providing a general picture of expression patterns under various conditions. Overall, the transcription of more than 20% (71 out of 333) of the intact LTR retroelements was detected in the *D. pulex *genome. For the purpose of comparison, we retrieved the expression pattern for 136 intact non-LTR retroelements that were identified in the *D. pulex *genome [32], and found that only eight (~5%) elements showed transcriptional activity and one of them had significantly long TARs (1138 bp). Additionally, we collected tiling array data for *D. melanogaster *at different developmental stages from the ENCODE website (Additional file 1 Table S5) and matched the TARs with the annotated LTR retroelements. In total, 25 (out of 412) intact elements from 12 families match with TARs, including 3 BEL, 1 *copia*, and 21 *gypsy *elements. Four elements from *roo *and *rover *families that have been shown to transpose previously [33,34], also showed transcriptional activity here (TAR length > 500 bp). The LTR retroelements in *D. pulex *exhibit higher transcriptional activity (in terms of the number and diversity of the elements) than those in *D. melanogaster*, even though there are fewer intact LTR retroelements identified in the *D. pulex *genome than those in the *D. melanogaster *genome.

Several elements in plant genomes are known to be able to transpose under specific conditions (e.g., high temperature [35,36]). Our study shows that the kairomone-exposed *Daphnia *show higher TE transcription levels than controls. Notably, under the same condition, the protein-coding genes of *Daphnia *also showed an overall higher transcription level, implying that global transcription activity is induced under the kairomone-exposed condition. On the other hand, the transcription level of LTR retroelements is not significantly different in the experiments comparing female vs. male and metal exposure. Although our analysis shows general trends in transcriptional activity, further experiments are required to investigate the activity of individual LTR retroelement families.

### TEs as components of the dynamic genome

Although no germline gains were observed in the mutation-accumulation lines, evidence for putative somatic gains was observed in both DIRS families assayed, providing additional evidence that there may be active retroelements in the *D. pulex *genome. The higher rate of putative somatic gains observed in lines in which sex occurred for the *Dpul_D15 *family is the opposite of the trend observed in DNA transposon families (Schaack et al. *accepted*). In addition to gains, lineages undergoing sex exhibited frequent losses for one family assayed, presumably because this family included heterozygotic copies (presence-absence) at the beginning of the experiment, which subsequently were lost 25% of the time via independent assortment of chromosomes during sex (which in this case was selfing). This difference highlights the importance of reproductive mode for the accumulation of mutation loads in the genome. Sexually-reproducing organisms can purge deleterious mutations (such as TE insertions) during recombination. Asexuals cannot purge TE insertions (other than via mitotic recombination at heterozygotic loci). As asexuals accumulate new mutations over time (Muller's ratchet [37]), it is thought that their fitness will decline and eventually they will go extinct [38].

Although the results of the transposon display assay support the idea that TEs may build up in asexual lineages over time, the data from the natural isolates indicate that, in nature, sexual isolates build up higher TE loads than asexuals, at least in one of the two families assayed (*Dpul_D5*). This result corroborates previous studies in *D. pulex *on the DNA transposon *Pokey *assayed among natural populations [39,40]. The increased number of TEs in sexuals could be explained in a number of ways. First, despite the increased efficiency of selection in sexual lineages, sex is a good way for new TE copies to spread among lineages in a population (whereas a new insertion in an asexual lineage is, effectively, at a genetic dead end). It is also possible that TE copies in recombining genomic backgrounds are able to better evade host suppression mechanisms because there is a higher chance of meiotic recombination among TE copies and therefore the production of novel genotypes undetectable by co-evolved suppression mechanisms. Alternatively, recombination events among retroelements belonging to the same family may render individual copies inactive, leading to a build-up over time of inactive copies in sexual lineages which is less likely in asexuals. Lastly, obligate asexuals that are able to persist in nature may represent isolates that evolved from especially low load sexual lineages, thereby minimizing the so-called "lethal hangover" from their sexual ancestors [41].

## Conclusions

We have performed a genome-wide analysis of the LTR retroelement content of the *D. pulex *genome, the first aquatic microcrustacean and cyclical parthenogen for which such an analysis has been performed. We identified 333 intact LTR retroelements in the *D. pulex *genome, and categorized them into BEL/*Pao, copia*, DIRS, and *gypsy *groups, respectively. As with other insects such as *D. melanogaster *and *A. gambiae*, the major group of retroelements in the *Daphnia *genome is *gypsy*, which includes almost half of the intact retroelements identified in this study. Notably, a very significant number of intact *copia *retroelements were identified as well. In addition, the *D. pulex *genome has been found to house the most DIRS elements among the arthropod genomes sequenced to date.

Transcriptional activity of intact LTR retroelements was surveyed by using tiling array data across the whole genome sequence. A total of 71 LTR retroelements showed expression signals, among which 12 elements contain long TAR regions. Transposon display assays of two intact DIRS retroelements were also performed and provide evidence for possible activity in mutation-accumulation lines of *D. pulex*. Patterns of TE load and polymorphism in natural populations indicate sexually-reproducing isolates have heavier TE loads and higher insertion site polymorphism among isolates for one family. Consistent with previously identified DIRS elements in fish and other animals, the *Daphnia *DIRS elements assayed here exhibit different structures of IR and protein domains (e.g., the YRs), compared with the elements from the other three groups. Further investigation of population-level differences for other families identified in this survey will help pinpoint which families of LTR retroelements remain active in the *D. pulex *genome and the extent to which they may influence genome evolution in this species.

## Methods

### Genomic sequences

The genomic sequences of *A. gambiae*, *B. mori*, *D. melanogaster*, *D. pulex*, and *O. sativa *genomes were obtained from public databases. The genomic sequence of *B. mori *(SW_scaffold_ge2k), *D. pulex *(release 1, jgi060905), and *O. sativa *(Build 4) were downloaded from VectorBase http://www.vectorbase.org, silkDB http://silkworm.genomics.org.cn, wFleaBase http://wFleaBase.org, JGI Genome Portal http://www.jgi.doe.gov/Daphnia/ and IRGSP http://rgp.dna.affrc.go.jp, respectively. The genomic sequence of *A. gambiae *(anoGam1) and *D. melanogaster *(dm3) were downloaded from UCSC Genome Bioinformatics site http://genome.ucsc.edu.

The RT sequences used in the phylogenetic analysis were obtained from NCBI web site: *BEL12 *(CAJ14165), *BEL *(U23420), *copia *(X04456), *GATE *(CAA09069), *Cer1 *(U15406), *Gulliver *(AF243513), *Mag *(X17219)*, gypsy *(X03734), *TED *(M32662)*, Yoyo *(U60529)*, Zam *(AJ000387)*, Tom *(Z24451)*, Tv1 *(AF056940)*, mdg1 *(X59545)*, 412 *(CAA27750)*, CsRn1 *(AAK07487)*, Kabuki *(BAA92689)*, Woot *(U09586)*, Osvaldo *(AJ133521)*, Blastopia *(CAA81643)*, mdg3 *(T13798)*, Cyclops *(AB007466)*, Maggy *(D18348), Ninja (AB043239), *Pao *(L09635), *Sushi *(AF030881), *Suzu *(AAN15112)*, 1731*(X07656), Hopscotch (T02087), Fourf (AAK73108).

Tiling array experiment results were collected form ENDCODE website http://intermine.modencode.org. The file name and DCCids are listed in Additional file 1 Table S5.

### Identification of intact LTR retroelements

We applied an automatic computational tool [11] to find intact LTR retroelements in the whole genome sequences listed above. The method in this study was improved to locate the TSDs and flanking ends of LTRs. Since it is not necessary for all intact LTR retroelements to have these features, we modified the program to be flexible by making this information optional. For example, although the majority of LTR flanking regions are di-nucleotides TG/CA, the well-known family *DM297 *in the *D. melanogaster *genome has di-nucleotides AG/CT. In the next step, the identified LTR retroelements were clustered into families based on the sequence similarity of LTRs between elements (sequence similarity > 80% for clustering elements in a family). Finally, the classified families were verified by using multiple sequence alignment of LTRs and IRs.

The element name consists of four parts: genome name, family name, scaffold name (release 1 from wfleaBase), and the ID in each scaffold. For example, the element *Dpul_G2_147_2 *corresponds to the second element in scaffold 147, which is in the family G2 (G for *gypsy *elements, C for *copia *elements, B for BEL element, and D for DIRS element) in the *D. pulex *genome.

### Phylogenetic analysis

For phylogenetic analysis, representative RT sequences were obtained from NCBI (see Materials and Methods section and Additional file 1 Table S6). Multiple sequence alignments of RT amino acid sequences were performed with default parameters by using CLUSTALW [42]. Phylogenetic trees were generated by using neighbor-joining tree method with poisson correction and 1000 bootstrap replicates in MEGA [43].

### Identification of LTR retroelement activity in mutation-accumulation lines

Mutation-accumulation lines were initiated in August 2004, from offspring originating from a single individual of *D. pulex *(Log50) originally collected from western Oregon. Log50 is the same isolate that was used for the genome sequencing project, and is from a relatively highly inbred, homozygous population. Lines were propagated by single-progeny each generation soon after their first clutch (~12 days at 20°C). Asexual lines were propagated by transferring either one or five (alternating each generation) random 1- to 2-day-old live female offspring to a new beaker. Crowding was used to generate cues inducing meiosis and when females produced males, selfed, and released resting eggs (ephippia), the eggs were collected and stored in tissue culture plates with 5-10 ml H_2_0 per well at 4°C. Resting eggs were typically produced 4-5 days after asexually-produced young had been born and transferred to a new beaker to propagate the original asexual line. Any ephippia that hatched after exposing eggs to short, intermittent periods of warmer temperatures (20°C) were used to initiate sexual sublines of asexual lineages. Sexual sublines (identified by their source asexual lineage and the generation at which the bout of sexual reproduction had occurred) were occasionally induced to reproduce sexually a second time, although only three such lineages were included in this survey. Other than hatching (and the conditions immediately preceding hatching), sexual sublines were maintained in the same manner over the course of the experiment as asexuals and treatments differ only by the occurrence of at least one (and occasionally two) bouts of sex. LTR retroelement activity was assayed in a subset of the mutation-accumulation lines (n = 93) after approximately 45 generations of single individual bottlenecks using transposon display. Transposon display (TD; [44]) was performed by using ECOR1 to digest genomic DNA extracted from 5-10 individuals from each lineage using CTAB (cetyltrimethylammonium bromide) extraction protocols adapted for *D. pulex *[45]. Digests were performed for 6 hrs at 37°C followed by 22 min at 80°C. Adaptors consisting of ~50 bp oligonucleotide pairs with a non-complementary mid-portion were ligated on to the ends of each fragment after the digest (16 hr ligation using T4 ligase at room temperature). Element-containing fragments are amplified via nested PCR using an element-specific primer (forward) and a reverse primer complementary to the non-complementary mid-portion of the ligated adaptors (Table 5). Only fragments of the genome containing copies of a given element amplify during PCR because the reverse primer cannot anneal unless the element-specific primer binds and elongates. This technique is sensitive but provides a lower-bound estimate for activity levels because long fragments may not amplify due to PCR bias. Conditions for the first and second round of PCR were as follows: initial denaturation at 94°C for 3 min, followed by 24 cycles of denaturation at 94°C for 30 sec, annealing at 5°C below the melting temperature for the element-specific primer, and elongation at 72°C for 1 min, and ending with a 5 min elongation step at 72°C. The second round of PCR used an element-specific primer slightly more towards the 3' end of the conserved region of the element. Since all lines were initiated from a single common ancestor, differences in banding pattern among descendent lineages indicate loss and/or gain of copies of individual elements within the genome. Peaks were scored as present if observed in multiple replicates (all assays were performed three times from the same ligated sample of DNA) and peaks that were above thresholds for inclusion but not observed in multiple replicates were scored as putative somatic insertions. The reason a non-replicable peak that was above threshold is considered a putative somatic insertion is because, given the DNA was extracted from 5-10 individuals, if an insertion occurred in somatic tissue, it would not be universally present in all three replicates. With only three replicates, this method most likely underestimates the frequency of somatic insertions, but can be used to detect a difference among treatments.

**Table 5:** Primer sequences for transposon display of *D. pulex *retroelements.

Oligos	Family	Primary primer	Secondary primer
Primers	*Dpul_D16*	CGTTAAGCCAGACCCACAGT	AGCGCCATCTCTCACCTATC
	*Dpul_D5*	AGGCTACGGCCTTCAGTTTT	TCTCTCTCATTCCTTCCTTG
	Reverse primer	GTAGACTGCGTACCAATTC	
Adaptors	Adaptor Top	CTCGTAGACTGCGTACC	
	Adaptor Bottom	AATTGGTACGCAGTCTAC	

## List of abbreviations

TE: Transposable element; LTR: Long terminal repeat; TSD: Target site duplication; RT: Reverse transcriptase; PR: Protease; YR: Tyrosine recombinase; IR: Internal region; ORF: Open reading frame, TAR: Transcriptionally active region.

## Authors' contributions

MR, SS, HT, ML and SK conceived the project. MR and XG conducted the data analysis. SS conducted the transposon display experiments. MR, SS and HT wrote the draft of the paper. All authors have read and approved the manuscript.

## Acknowledgements

We would like to thank Dr. John Colbourne and Dr. Jeong-Hyeon Choi for helpful discussion and allowing us to access tiling array data. We thank Dr. Ellen Pritham for reading the manuscript and helpful discussion. This work is supported by MetaCyt Initiative at Indiana University, funded by Lilly Endowment, Inc. It is also supported by NSF DDIG (DEB-0608254) to SS and ML, NIH training grant fellowship to SS, and NIH fellowship F32GM083550 to XG. The sequencing and portions of the analyses were performed at the DOE Joint Genome Institute under the auspices of the U.S. Department of Energy's Office of Science, Biological and Environmental Research Program, and by the University of California, Lawrence Livermore National Laboratory under Contract No. W-7405-Eng-48, Lawrence Berkeley National Laboratory under Contract No. DE-AC02-05CH11231, Los Alamos National Laboratory under Contract No. W-7405-ENG-36 and in collaboration with the Daphnia Genomics Consortium (DGC) http://daphnia.cgb.indiana.edu. Additional analyses were performed by wFleaBase, developed at the Genome Informatics Lab of Indiana University with support to Don Gilbert from the National Science Foundation and the National Institutes of Health. Coordination infrastructure for the DGC is provided by the Center for Genomics and Bioinformatics at Indiana University, which is supported in part by the METACyt Initiative of Indiana University, funded in part through a major grant from the Lilly Endowment, Inc. Our work benefits from, and contributes to the Daphnia Genomics Consortium.

## Supplementary Material

Additional file 1 **The file includes four tables**. Table S1. Intact LTR retroelements in the *D. pulex *genome. Table S2. Summary of intact LTR retroelements in five genomes. Table S3. Summary of TEs transcribed under three different conditions. Table S4. A list of LTR retroelements expressed in tiling array data. Table S5. A list of tiling array information collected ENCODE website Table S6: A list of RT sequences in Figure 1. Click here for file
