# Supplementary material for: LTR retroelements in the genome of Daphnia pulex
Source: BMC Genomics. 2010 Jul 9;11:425. doi: 10.1186/1471-2164-11-425 (PMC2996953; doi:10.1186/1471-2164-11-425)
Supplement: Additional file 1 — The file includes four tables. Table S1. Intact LTR retroelements in the D. pulex genome. Table S2. Summary of intact LTR retroelements in five genomes. Table S3. Summary of TEs transcribed under three different conditions. Table S4. A list of LTR retroelements expressed in tiling array data. Table S5. A list of tiling array information collected ENCODE website Table S6: A list of RT sequences in Figure 1. [file 1471-2164-11-425-S1.DOC]

Table S1. Intact LTR retroelements in the *D. pulex* genome.

| Family | LTR  Sim | LTR  length | Element  length | Copy # | Avg. sim^*^ |  | Family | LTR  Sim | | LTR  length | Element length | Copy # | Avg. sim^*^ |
| --- | --- | --- | --- | --- | --- | --- | --- | --- | --- | --- | --- | --- | --- |
| Dpul_B1 | 95.0 | 252 | 6578 | 2 | 77.70 |  | Dpul_C38 | | 90.5 | 255 | 8184 | 3 | 58.56 |
| Dpul_B2 | 96.0 | 499 | 7408 | 3 | 44.93 |  | Dpul_C39 | | 100.0 | 288 | 4560 | 1 | - |
| Dpul_B3 | 96.7 | 414 | 6779 | 3 | 88.10 |  | Dpul_C40 | | 99.5 | 186 | 4558 | 1 | - |
| Dpul_B4 | 96.9 | 329 | 7833 | 10 | 56.09 |  | Dpul_C41 | | 98.3 | 360 | 4805 | 1 | - |
| Dpul_B5 | 98.5 | 373 | 7677 | 2 | 70.50 |  | Dpul_C42 | | 98.7 | 313 | 4700 | 1 | - |
| Dpul_B6 | 99.5 | 421 | 8142 | 3 | 77.40 |  | Dpul_C43 | | 99.4 | 321 | 5390 | 4 | 71.20 |
| Dpul_B7 | 96.5 | 219 | 7473 | 2 | 53.60 |  | Dpul_C44 | | 99.4 | 318 | 4644 | 1 | - |
| Dpul_B8 | 98.4 | 247 | 7779 | 2 | 72.30 |  | Dpul_G1 | | 98.0 | 396 | 9939 | 4 | 64.06 |
| Dpul_B9 | 99.8 | 413 | 5907 | 1 | - |  | Dpul_G2 | | 99.0 | 185 | 5072 | 15 | 71.70 |
| Dpul_B10 | 99.5 | 558 | 5705 | 2 | 48.30 |  | Dpul_G3 | | 83.6 | 165 | 4867 | 1 | - |
| Dpul_B11 | 99.0 | 494 | 9235 | 3 | 82.20 |  | Dpul_G4 | | 95.6 | 329 | 5889 | 2 | 82.00 |
| Dpul_B12 | 98.5 | 467 | 8412 | 4 | 66.51 |  | Dpul_G5 | | 98.4 | 220 | 6981 | 3 | 56.43 |
| Dpul_B13 | 100.0 | 237 | 12536 | 1 | - |  | Dpul_G6 | | 98.2 | 938 | 10547 | 2 | 71.40 |
| Dpul_B14 | 98.8 | 713 | 6499 | 3 | 83.30 |  | Dpul_G7 | | 98.8 | 367 | 7055 | 14 | 59.65 |
| Dpul_B15 | 95.2 | 267 | 9106 | 5 | 54.46 |  | Dpul_G8 | | 98.7 | 639 | 11164 | 13 | 56.41 |
| Dpul_B16 | 99.2 | 1735 | 10248 | 2 | 55.00 |  | Dpul_G9 | | 99.3 | 134 | 4026 | 1 | - |
| Dpul_B17 | 97.1 | 438 | 6339 | 4 | 63.20 |  | Dpul_G10 | | 98.6 | 230 | 6309 | 4 | 52.30 |
| Dpul_B18 | 99.4 | 307 | 8372 | 3 | 70.10 |  | Dpul_G11 | | 96.7 | 206 | 5056 | 10 | 49.58 |
| Dpul_B19 | 99.2 | 227 | 6767 | 2 | 99.10 |  | Dpul_G12 | | 96.6 | 633 | 10862 | 5 | 49.9 |
| Dpul_B20 | 88.9 | 409 | 6030 | 1 | - |  | Dpul_G13 | | 98.8 | 195 | 5310 | 2 | 71.30 |
| Dpul_B21 | 99.0 | 193 | 7379 | 1 | - |  | Dpul_G14 | | 97.6 | 374 | 10497 | 1 | - |
| Dpul_B22 | 98.6 | 668 | 6447 | 2 | 82.10 |  | Dpul_G15 | | 97.2 | 318 | 5842 | 3 | 82.01 |
| Dpul_B23 | 100.0 | 498 | 6685 | 1 | - |  | Dpul_G16 | | 96.7 | 222 | 9442 | 2 | 50.60 |
| Dpul_B24 | 99.5 | 610 | 6541 | 2 | 73.10 |  | Dpul_G17 | | 100.0 | 172 | 5452 | 1 | - |
| Dpul_B25 | 97.4 | 227 | 3349 | 1 | - |  | Dpul_G18 | | 99.0 | 163 | 6968 | 3 | 61.63 |
| Dpul_B26 | 98.4 | 254 | 5872 | 1 | - |  | Dpul_G19 | | 99.3 | 286 | 9912 | 2 | 44.20 |
| Dpul_C1 | 99.1 | 251 | 4971 | 4 | 81.93 |  | Dpul_G20 | | 99.1 | 212 | 4834 | 1 | - |
| Dpul_C2 | 98.9 | 274 | 4451 | 1 | - |  | Dpul_G21 | | 100.0 | 323 | 5499 | 2 | 99.80 |
| Dpul_C3 | 96.2 | 244 | 4438 | 3 | 61.30 |  | Dpul_G22 | | 94.6 | 446 | 5617 | 1 | - |
| Dpul_C4 | 97.5 | 279 | 6574 | 3 | 49.53 |  | Dpul_G23 | | 98.6 | 443 | 6427 | 1 | - |
| Dpul_C5 | 99.5 | 361 | 5337 | 2 |  |  | Dpul_G24 | | 99.3 | 149 | 4436 | 4 | 63.85 |
| Dpul_C6 | 97.9 | 424 | 4937 | 3 | 71.06 |  | Dpul_G25 | | 97.6 | 185 | 4898 | 3 | 86.06 |
| Dpul_C7 | 97.1 | 249 | 5861 | 6 | 68.85 |  | Dpul_G26 | | 99.6 | 459 | 8358 | 4 | 69.30 |
| Dpul_C8 | 99.0 | 298 | 4984 | 4 | 68.96 |  | Dpul_G27 | | 99.3 | 190 | 6139 | 3 | 82.76 |
| Dpul_C9 | 99.7 | 519 | 4456 | 3 | 48.53 |  | Dpul_G28 | | 98.8 | 366 | 6835 | 5 | 62.97 |
| Dpul_C10 | 98.6 | 234 | 5524 | 2 | 54.6 |  | Dpul_G29 | | 99.3 | 882 | 8666 | 2 | 45.00 |
| Dpul_C11 | 100.0 | 172 | 4355 | 1 | - |  | Dpul_G30 | | 97.1 | 419 | 9656 | 1 | - |
| Dpul_C12 | 99.2 | 602 | 4972 | 3 | 92.50 |  | Dpul_G31 | | 99.6 | 447 | 12862 | 2 | 92.60 |
| Dpul_C13 | 99.8 | 292 | 5814 | 10 | 49.27 |  | Dpul_G32 | | 99.0 | 386 | 7413 | 6 | 53.08 |
| Dpul_C14 | 99.2 | 256 | 4400 | 1 | - |  | Dpul_G33 | | 98.4 | 372 | 6073 | 1 | - |
| Dpul_C15 | 95.2 | 212 | 4640 | 2 | 76.00 |  | Dpul_G34 | | 98.5 | 166 | 4959 | 2 | 85.20 |
| Dpul_C16 | 99.7 | 366 | 4577 | 2 | 80.30 |  | Dpul_G35 | | 93.0 | 898 | 10335 | 2 | 54.70 |
| Dpul_C17 | 92.5 | 299 | 5183 | 1 | - |  | Dpul_G36 | | 100.0 | 169 | 4840 | 1 | - |
| Dpul_C18 | 98.1 | 339 | 4964 | 3 | 66.83 |  | Dpul_G37 | | 100.0 | 638 | 7999 | 2 | 60.8 |
| Dpul_C19 | 99.1 | 323 | 4804 | 1 | - |  | Dpul_G38 | | 100.0 | 158 | 5101 | 1 | - |
| Dpul_C20 | 99.6 | 249 | 5400 | 1 | - |  | Dpul_G39 | | 98.8 | 168 | 6730 | 3 | 40.2 |
| Dpul_C21 | 98.6 | 218 | 4528 | 1 | - |  | Dpul_G40 | | 97.8 | 181 | 8088 | 1 | - |
| Dpul_C22 | 99.9 | 314 | 4797 | 2 | 99.40 |  | Dpul_G41 | | 99.3 | 757 | 10446 | 1 | - |
| Dpul_C23 | 99.1 | 218 | 5445 | 2 | 54.00 |  | Dpul_G42 | | 92.0 | 391 | 6463 | 1 | - |
| Dpul_C24 | 92.9 | 301 | 4755 | 2 | 74.10 |  | Dpul_G43 | | 95.8 | 185 | 5652 | 2 | 82.2 |
| Dpul_C25 | 98.8 | 323 | 6567 | 1 | - |  | Dpul_G44 | | 99.3 | 410 | 5868 | 1 | - |
| Dpul_C26 | 97.0 | 227 | 4763 | 2 | 93.70 |  | Dpul_G45 | | 88.7 | 190 | 9895 | 1 | - |
| Dpul_C27 | 99.5 | 369 | 4704 | 1 | - |  | Dpul_G46 | | 98.3 | 647 | 10641 | 1 | - |
| Dpul_C28 | 92.7 | 252 | 5596 | 1 | - |  | Dpul_G47 | | 99.8 | 551 | 11381 | 1 | - |
| Dpul_C29 | 97.9 | 254 | 7958 | 2 | 47.10 |  | Dpul_G48 | | 98.2 | 717 | 9382 | 1 | - |
| Dpul_C30 | 100.0 | 190 | 5267 | 1 | - |  | Dpul_G49 | | 87.2 | 374 | 6431 | 1 | - |
| Dpul_C31 | 100.0 | 218 | 4488 | 1 | - |  | Dpul_G50 | | 99.3 | 394 | 7059 | 2 | 90.30 |
| Dpul_C32 | 100.0 | 209 | 4064 | 2 | 83.30 |  | Dpul_G51 | | 98.6 | 148 | 5696 | 1 | - |
| Dpul_C33 | 100.0 | 203 | 4465 | 3 | 79.23 |  | Dpul_G52 | | 96.9 | 162 | 4807 | 1 | - |
| Dpul_C34 | 99.9 | 356 | 6863 | 2 | 64.50 |  | Dpul_G53 | | 94.6 | 168 | 5458 | 1 | - |
| Dpul_C35 | 99.0 | 291 | 4798 | 1 | - |  | Dpul_G54 | | 83.8 | 287 | 4294 | 1 | - |
| Dpul_C36 | 98.9 | 190 | 5927 | 1 | - |  | Dpul_G55 | | 99.7 | 391 | 8629 | 1 | - |
| Dpul_C37 | 99.1 | 279 | 4763 | 2 |  |  | Dpul_G56 | | 98.2 | 493 | 11209 | 3 | 83.33 |

Table S2. Summary of intact LTR retroelements in five genomes.

| Genome | BEL | *copia* | DIRS | *gypsy* | Total |
| --- | --- | --- | --- | --- | --- |
| *A. gambiae* | 161 (49) | 22 (7) | - | 166 (69) | 349 (125) |
| *B. mori* | 8 (7)  7290(7) ^a^ | 3 (3)  5585(4) ^a^ | - | 19 (17)  16152(13) ^a^ | 30 (27) |
| *D. melanogaster* | 114 (7)  71(7)^b^ | 30 (3)  28(4) ^b^ | - | 268 (31)  205(34) ^b^ | 412 (41) |
| *D. pulex* | 66 (26) | 95 (44) | 19 (15) | 153 (56) | 333 (141) |
| *O. sat* | - | 698 (108)  4350(24) ^c^ | - | 1381 (117)  7050(20) ^c^ | 2079 (225) |

*The number in parentheses represents the number of families.

^a^ The number of LTR retroelements (intact and fragmented) surveyed in ref. [21].

^b^ The number of intact LTR retroelements surveyed in ref [3].

^c^ The number of LTR retroelements (intact and fragmented) surveyed in ref. [18].

Table S3. A list of LTR retroelements expressed in tiling array data.

| Column Number | Element ^1^ | Start position^2^ |
| --- | --- | --- |
| 1  2  3  4  5  6  7  8  9  10  11  12  13  14  15  16  17  18  19  20  21  22  23  24  25  26  27  28  29  30  31  32  33  34  35  36  37  38  39  40  41  42  42  44  45  46  47  48  49  50  51  52  53  54  55  56  57  58  59  60  61  62  63  64  65  66  67  68  69  70  71 | Dpul_B17_ 22  Dpul_B19_37  Dpul_B21_30  Dpul_B24_69  Dpul_B25_48  Dpul_B4_57  Dpul_C1_1381  Dpul_C13_27  Dpul_C14_154  Dpul_C15_161  Dpul_C17_175  Dpul_C19_205  Dpul_C23_271  Dpul_C28_260  Dpul_C30_31  Dpul_C31_34  Dpul_C33_35  Dpul_C33 _66  Dpul_C36 _44  Dpul_C38 _54  Dpul_C40 _71  Dpul_C41 _728  Dpul_C43 _883  Dpul_C7 _115  Dpul_C7 _836  Dpul_C8 _118  Dpul_C8 _26  Dpul_C8 _272  Dpul_C8 _40  Dpul_D2_108  Dpul_D14_58  Dpul_G1 _153  Dpul_G1 _57  Dpul_G11 _309  Dpul_G12 _140  Dpul_G12 _82  Dpul_G13 _137  Dpul_G16 _847  Dpul_G19 _26  Dpul_G23 _192  Dpul_G24 _66  Dpul_G25 _201  Dpul_G27 _33  Dpul_G28 _33  Dpul_G28 _35  Dpul_G31 _22  Dpul_G31 _49  Dpul_G32 _116  Dpul_G32 _177  Dpul_G32 _22  Dpul_G32 _744  Dpul_G34 _92  Dpul_G36 _27  Dpul_G39 _50  Dpul_G40 _37  Dpul_G41 _37  Dpul_G42 _41  Dpul_G45 _50  Dpul_G5 _108  Dpul_G5 _96  Dpul_G52 _64  Dpul_G53 _73  Dpul_G54 _79  Dpul_G55 _92  Dpul_G56 _172  Dpul_G56 _66  Dpul_G7 _112  Dpul_G7 _169  Dpul_G7 _50  Dpul_G7 _54  Dpul_G8 _201 | 1152678  842365  1095294  303970  724286  85580  1342  426132  222036  267301  143408  69680  106444  73082  930571  1007251  412714  34319  491532  633248  404089  4568  105  294526  2600  81961  1060039  25602  919844  387059  703677  97625  553446  23183  135289  218914  33706  4095  909981  140539  136986  52465  160965  170042  685776  74408  697716  192518  135535  1023996  1039  101174  603017  308351  88131  801722  180087  710645  185367  58110  742385  311622  417285  175093  98397  320517  285221  162521  500730  481247  131830 |

^1^ Elements are named by organism name, family name, and scaffold name.

^2^ Start position (bp) is in the genomic sequence of scaffold.

Table S4. Summary of TEs transcribed under three different conditions.

| Condition | # elements expressed | # elements exclusively expressed in this condition | Avg. expression level |
| --- | --- | --- | --- |
| Female | 39 | 2 | 2.45 |
| Male | 42 | 6 | 1.47 |
| Control for Metal exposure | 26 | 6 | 1.24 |
| Metal exposure | 21 | 1 | 1.72 |
| Control for kairomone exposure | 8 | 1 | 1.30 |
| Kairomone exposure | 22 | 4 | 1.96 |

Table S5. A list of RT sequences in Figure 1.

| Sequence name | Organism | Element name |
| --- | --- | --- |
| rice_C48_chr02_54  rice_C83_chr06_272  rice_C13_chr01_19  rice_C43_chr02_21  rice_C89_chr07_272  rice_C47_chr02_34  rice_C38_chr02_17  rice_C18_chr01_253  rice_C58_chr03_82  rice_C28_chr01_311  rice_C4_chr01_108  D_DIRS-1  D_Pat  D_TnDirs  D_Drdirs2  D_Drdirs3  D_Spdirs4  D_LvDirs1  D_Tcdirs1  D_spdirs1  D_spdirs3  D_spdirs2  D_Rodirs1  B_Cer13_y75d11a  B_Cer8_zk262  B_Cer9_y43f4a  B_Cer10_y81b8a  B_Cer12_f21d9  B_Cer10_t23b12  B_Cer11_t14g12  B_Cer7_zc132  B_Cer15_y102a5d  B_Cer9_w09b7  B_BEL_DM  B_Pao_BM  B_Suzu_Frub  B_CATCH1_Frub  B_Kamikaze  B_SeaUrchin  B_BEL12_AG  B_GATE_DM  B_Moose_AG  B_Ninja_DS  C_1731_DM  C_Copia_X04456_DM  C_Hopscotch_U12626_ZM  C_Mosqcopia_AA  C_RIRE1_RT_C  C_Ty1  C_Ty4  C_Ty5-6p  G_297_DM_CAB57796  G_Gypsy_DM_AAA70219  G_412_DM_CAA27750  G_mdg1_DM_X59545  G_Ty3_CAA97115_SC  G_mdg3_DM_CAA65152  G_Blastopia_CAA81643_DM  G_Osvaldo_AJ133521_DB  G_CSRN1_AAK07487_CS  G_Mag_BM_S08405  G_Maggy_AAA33420_MG  G_SURL_M75523_TG  G_TED_TN_AAA92249  G_Sushi_AF030881_FR | O.sativa  O.sativa  O.sativa  O.sativa  O.sativa  O.sativa  O.sativa  O.sativa  O.sativa  O.sativa  O.sativa  D.discoideum  P.redivivus  T.nigroviridis  D.rerio  D.rerio  S.purpuratus  L.variegatus  T.castaneum  S.purpuratus  S.purpuratus  S.purpuratus  R.oryzae  C.elegans  C.elegans  C.elegans  C.elegans  C.elegans  C.elegans  C.elegans  C.elegans  C.elegans  C.elegans  D.melanogaster  B.mori  T.rubripes  T.rubripes  B.mori  S.purpuratus  A.gambiae  D.melanogaste  A.gambiae  D.simulans  D.melanogaste  D.melanogaste  Z.mays  A.aegypti  O.australiensis  S.cerevisiae  S.cerevisiae  S.cerevisiae  D.melanogaster  D.melanogaster  D.melanogaster  D.melanogaster  S.cerevisiae  D.melanogaster  D.melanogaster  D.buzzatii  C.sinensis  B.mori  M.grisea  T.gratilla  Autographa  T.rubripes | Osat_C48 (this study)  Osat_C83 (this study)  Osat_C13 (this study)  Osat_C43 (this study)  Osat_C89 (this study)  Osat_C47 (this study)  Osat_C38 (this study)  Osat_C18 (this study)  Osat_C58 (this study)  Osat_C28 (this study)  Osat_C4 (this study)  DIRS-1  PAT  TnDirs  Drdirs2  Drdirs3  Spdirs4  LvDirs1  Tcdirs1  spdirs1  spdirs3  spdirs2  Rodirs1  Cer13  Cer8  Cer9  Cer10  Cer12  Cer10  Cer11  Cer7  Cer5  Cer9  BEL  Pao  Suzu  CATCH1  Kamikaze  BEL12  GATE  Moose  Ninja  1731  Copia  Hopscotch  Mosqcopia  RIRE1  Ty1  Ty4  Ty5  297  Gypsy  412  mdg1  Ty3  mdg3  Blastopia  Osvaldo  CSRN1  Mag  Maggy  SURL  californica  Sushi |

Table S6. A list of tiling array information collected ENCODE website

| Title | DCCid |
| --- | --- |
| Dm_y[1]cn[1] sp[1]_adult_Female_1dayPostEclosion_TotalRNA_p200_374-376-378_38bp  Dm_y[1]cn[1] sp[1]_adult_Female_5dayPostEclosion_TotalRNA_p200_386-388-390_38bp  Dm_y[1]cn[1] sp[1]_adult_Male_1dayPostEclosion_TotalRNA_p200_368-370-372_38bp  Dm_y[1]cn[1] sp[1]_adult_Male_5dayPostEclosion_TotalRNA_p200_380-382-384_38bp  Dm_y[1]cn[1] sp[1]_embryo_0-2h_TotalRNA_p200_212-214-216_38bp  Dm_y[1]cn[1] sp[1]_embryo_10-12h_TotalRNA_p200_242-244-246_38bp  Dm_y[1]cn[1] sp[1]_embryo_12-14h_TotalRNA_p200_248-250-252_38bp  Dm_y[1]cn[1] sp[1]_embryo_14-16h_TotalRNA_p200_254-256-258_38bp  Dm_y[1]cn[1] sp[1]_embryo_16-18h_TotalRNA_p200_260-262-264_38bp  Dm_y[1]cn[1] sp[1]_embryo_18-20h_TotalRNA_p200_266-268-270_38bp  Dm_y[1]cn[1] sp[1]_embryo_20-22h_TotalRNA_p200_274-276_38bp  Dm_y[1]cn[1] sp[1]_embryo_22-24h_TotalRNA_p200_278-280-282_38bp  Dm_y[1]cn[1] sp[1]_embryo_2-4h_TotalRNA_p200_218-220-222_38bp  Dm_y[1]cn[1] sp[1]_embryo_4-6h_TotalRNA_p200_224-226-228_38bp  Dm_y[1]cn[1] sp[1]_embryo_6-8h_TotalRNA_p200_230-232_38bp  Dm_y[1]cn[1] sp[1]_embryo_8-10h_TotalRNA_p200_236-238-240_38bp  Dm_y[1]cn[1] sp[1]_larval_L1_TotalRNA_p200_284-286-288_38bp  Dm_y[1]cn[1] sp[1]_larval_L2_TotalRNA_p200_290-292-294_38bp  Dm_y[1]cn[1] sp[1]_larval_L3_gut_stage1_TotalRNA_p200_440-442-444_38bp | 96  97  98  99  101  105  107  109  111  113  116  117  118  120  763  103  93  94  100 |
